# Rat Model for Dominant Dystrophic Epidermolysis Bullosa: Glycine Substitution Reduces Collagen VII Stability and Shows Gene-Dosage Effect

**DOI:** 10.1371/journal.pone.0064243

**Published:** 2013-05-23

**Authors:** Alexander Nyström, Jens Buttgereit, Michael Bader, Tatiana Shmidt, Cemil Özcelik, Ingrid Hausser, Leena Bruckner-Tuderman, Johannes S. Kern

**Affiliations:** 1 Department of Dermatology, University Freiburg Medical Center, Freiburg, Germany; 2 Max Delbück Center for Molecular Medicine (MDC), Campus Berlin-Buch, Berlin, Germany; 3 Experimental and Clinical Research Center (ECRC), Berlin, Germany; 4 Department of Dermatology, University of Heidelberg, Heidelberg, Germany; 5 Freiburg Institute for Advanced Studies, School of Life Sciences, LifeNet, University of Freiburg, Freiburg, Germany; University of Vienna, Max F. Perutz Laboratories, Austria

## Abstract

Dystrophic epidermolysis bullosa, a severely disabling hereditary skin fragility disorder, is caused by mutations in the gene coding for collagen VII, a specialized adhesion component of the dermal-epidermal junction zone. Both recessive and dominant forms are known; the latter account for about 40% of cases. Patients with dominant dystrophic epidermolysis bullosa exhibit a spectrum of symptoms ranging from mild localized to generalized skin manifestations. Individuals with the same mutation can display substantial phenotypic variance, emphasizing the role of modifying genes in this disorder. The etiology of dystrophic epidermolysis bullosa has been known for around two decades; however, important pathogenetic questions such as involvement of modifier genes remain unanswered and a causative therapy has yet to be developed. Much of the failure to make progress in these areas is due to the lack of suitable animal models that capture all aspects of this complex monogenetic disorder. Here, we report the first rat model of dominant dystrophic epidermolysis bullosa. Affected rats carry a spontaneous glycine to aspartic acid substitution, p.G1867D, within the main structural domain of collagen VII. This confers dominant-negative interference of protein folding and decreases the stability of mutant collagen VII molecules and their polymers, the anchoring fibrils. The phenotype comprises fragile and blister-prone skin, scarring and nail dystrophy. The model recapitulates all signs of the human disease with complete penetrance. Homozygous carriers of the mutation are more severely affected than heterozygous ones, demonstrating for the first time a gene-dosage effect of mutated alleles in dystrophic epidermolysis bullosa. This novel viable and workable animal model for dominant dystrophic epidermolysis bullosa will be valuable for addressing molecular disease mechanisms, effects of modifying genes, and development of novel molecular therapies for patients with dominantly transmitted skin disease.

## Introduction

Epidermolysis bullosa (EB) comprises a heterogeneous group of hereditary skin fragility disorders. So far, mutations in 18 genes are known to give rise to different forms of EB [1]–[3]. Based on the ultrastructural level of skin blistering, EB can be divided into four major types: EB simplex, junctional EB, dystrophic EB (DEB) and Kindler syndrome [4]; these main categories are further divided into subtypes. In addition to painful skin blisters and wounds, severely affected patients can have mucosal involvement, causing malnutrition. A common denominator for genes linked with EB is that they code for proteins, which, at least partially bear non-redundant structural functions vital for skin integrity.

One such protein is collagen VII (C7), a specialized component of the dermal-epidermal junction zone and major constituent of the anchoring fibrils that attach the epidermis to the dermis [3]. C7 is an atypical collagen with a large N-terminal globular non-collagenous domain (NC1), a central collagenous domain and another globular non-collagenous domain at the C-terminal end (NC2) [5]. Individual C7 monomers, composed of three identical α-chains, form antiparallel dimers, which aggregate into anchoring fibril suprastructures [5], [6]. Anchoring fibrils support skin stability by interacting with components of the dermal-epidermal basement membrane while wrapping around fibrillar collagens in the dermal extracellular matrix, thus anchoring the overlying basement membrane to the underlying dermis [7]. Mutations in the gene coding for C7, *COL7A1*, cause DEB [7], which accounts for approximately 25% of all EB cases worldwide (www.debra-international.org).

DEB manifests with fragile skin, blistering of the mouth, esophagus, and rectum, healing with excessive scarring, and nail dystrophy [8]. Depending on the mutation, DEB can be inherited in a dominant (DDEB) or recessive (RDEB) mode [8]. DDEB is generally regarded as less severe, whereas patients with severe generalized RDEB have the highest morbidity. DDEB accounts for about 40% of all diagnosed DEB cases [9] and has a disease spectrum that ranges from mild localized to generalized skin manifestations. One particularly intriguing aspect of DDEB is that the same mutation can give rise to variable phenotypes in different patients [8], [10], suggesting that environmental factors and modifier genes are of importance in the pathogenesis. However, lack of suitable models has hampered the study of such effects.

Several animal models of RDEB have been described, both engineered [11]–[14] and spontaneous [14]–[22]. Many of the spontaneous models are severe and lethal shortly after birth. In some cases, the large size of some animals renders them difficult to work with. As yet, no model exists for DDEB. Although an Akita dog was suggested to have DDEB [19], the mode of inheritance could not be conclusively determined.

In this study, we report a novel rat model of DDEB. Affected rats carry a glycine to aspartic acid substitution within the major structural (collagenous) domain of C7. The substitution decreases the stability of C7 monomers carrying one or more mutated α-chains, thus conferring dominant-negative interference. The rats have fragile and blister-prone skin as a consequence of fewer and thinner anchoring fibrils. The model recapitulates all signs of human DDEB with complete penetrance, but as in patients, the animals exhibit individual variance of the disease manifestations. Importantly, homozygous carriers of the mutation are more severely affected than heterozygous carriers, suggesting a gene-dosage effect of the mutation. This novel small animal model will be of value for addressing specific questions relating to molecular disease mechanisms in DDEB and development of novel molecular therapies.

## Results

### Clinical phenotype

**Figure 1:**
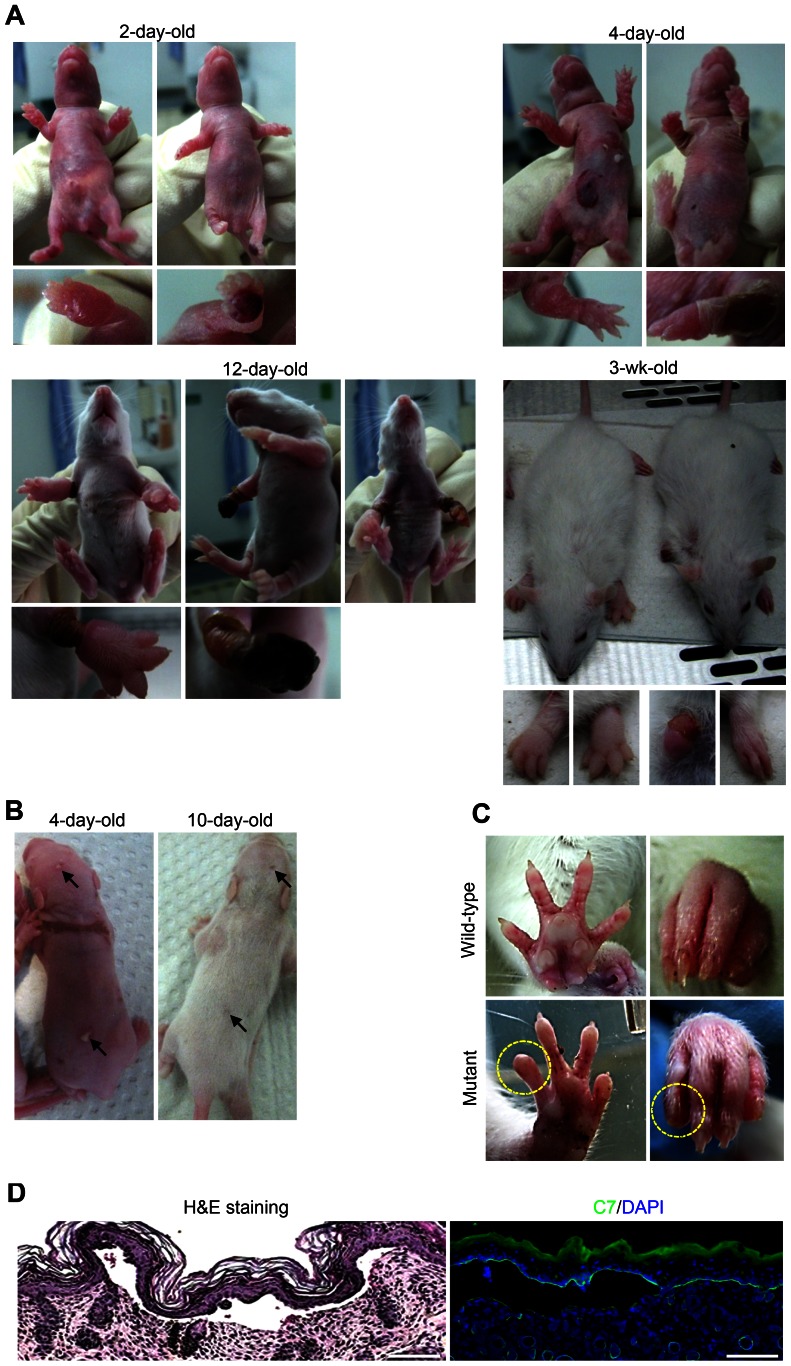
Clinical presentation of the spontaneous skin blistering phenotype in DEB rats. **A**, Progression of skin blistering from birth to adulthood. At birth, blistering is mainly restricted to the footpads. With time, it spreads to the belly and the chest, and large erosions occur on the limbs. With the beginning of fur growth – which leads to skin stabilization – blistering decreases. Poorly healing paws lead to mutilating deformities. **B**, Soon after birth (4–10 days), small blisters develop on the back skin; these heal with scarring (arrows). **C**, In adult rats the phenotype is mainly restricted to the paws; note the dystrophic claws or toes with lost claws (yellow circle). **E**, Histological analysis of the skin from newborn mutant rats shows separation of the epidermis from the dermis. These findings are compatible with DEB. Left panel: H&E staining. Right panel: C7 antibody staining reveals a signal at the blister roof. Scale bar = 100 µm.

Spontaneous skin blistering and erosions were observed in pups from inbred Sprague Dawley rats. Shortly after birth, affected pups developed blood-filled blisters on the extremities, the back and the head (Figure 1A). Over the next few days the phenotype grew progressively worse, with blistering expanding on the limbs and occurring on the chest and belly. Loss of large areas of skin was also regularly observed. Malnutrition was obvious in some rats (Figure 1A), suggesting mucosal blistering. In the most severe cases, the rats died in the second or third week of life.

With the beginning of fur growth – which leads to skin stabilization – blistering ceased, leaving some scarring (Figure 1B). Healing of affected paws varied greatly; some paws healed without visible signs of the previous blistering, whereas other paws remained ulcerated. This led to malformation and in the most severe cases mutilating deformities (Figure 1A). Adult rats with a history of postnatal blistering and subsequent healing exhibited dystrophy or loss of toes (Figure 1C).

The phenotype suggested a generalized form of EB with high severity. Histopathological analyses of skin biopsy specimens from affected rats revealed complete detachment of the epidermis from the dermis (Figure 1D) and immunomapping of the dermal-epidermal junction (DEJZ) demonstrated that blisters arose in the uppermost dermis. C7 staining remained at the blister roof (Figure 1D), indicating that the rats were affected with DEB.

### Electron microscopy

**Figure 2:**
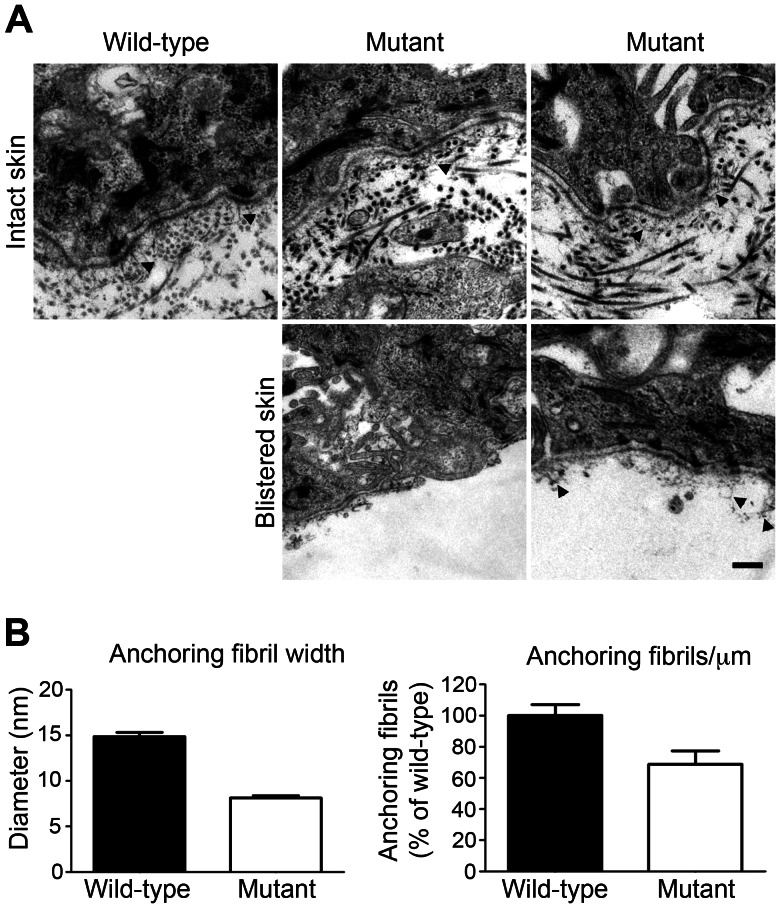
Electron microscopy demonstrates anchoring fibril abnormalities in mutant rat skin. **A**, Transmission electron microscopy images of the skin from four-day-old wild-type and mutant rats. Electron dense anchoring fibrils (arrowheads) are clearly visible under the lamina densa in wild-type rats, whereas mutant rat skin contains fewer and thinner anchoring fibrils (arrowheads). In blistered skin abnormal and collapsed anchoring fibrils are attached to the blister roof (arrowheads). Scale bar = 250 nm **B**, Quantification of the electron micrographs confirm the notion of fewer and thinner anchoring fibrils in mutant rat skin.

C7 is the major component of anchoring fibrils, which attach the epidermis to the underlying dermis. By electron microscopy, the anchoring fibrils were clearly visible as cross-banded fibrils at the DEJZ in the skin of age-matched wild-type rats (Figure 2A). In contrast, the fibrils appeared sparser and thinner in the skin of the mutant rats. Blistering occurred below the lamina densa level, and loose collapsed fibrils remained attached to the blister roof (Figure 2A). Quantification of images of randomly selected areas in electron micrographs confirmed the above notion, with anchoring fibrils in affected rats being both thinner and less abundant (Figure 2B).

### Mutation analysis

**Figure 3:**
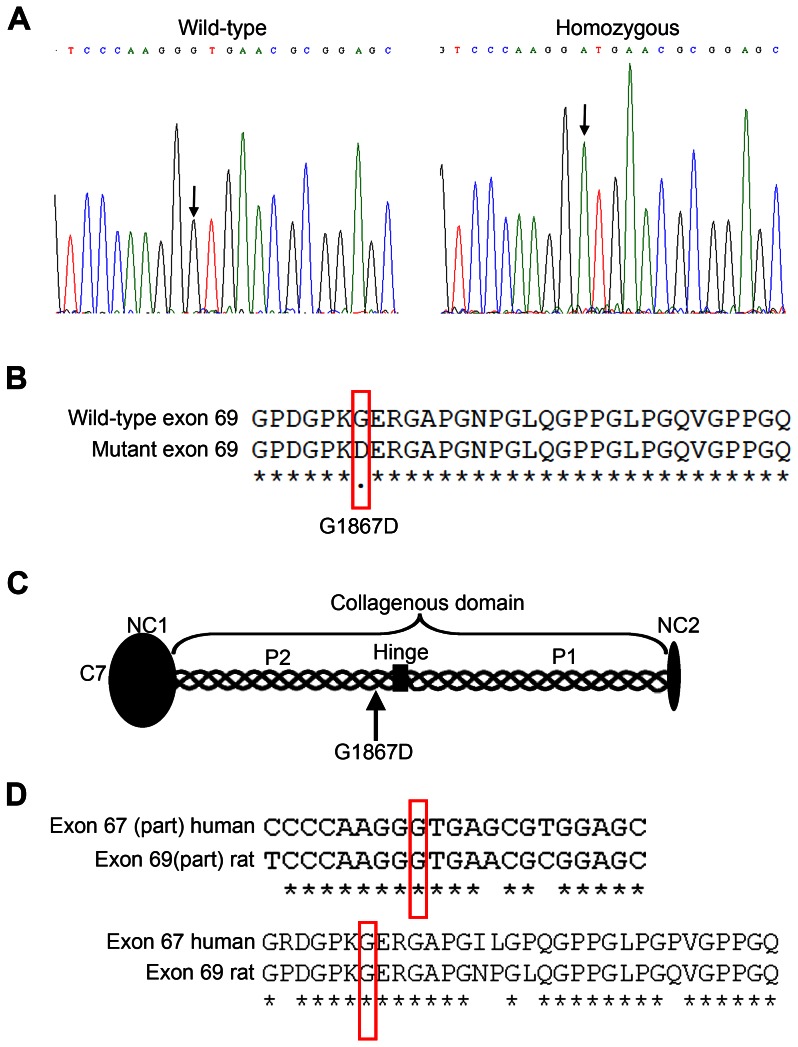
The mutant rats carry a *Col7a1* missense mutation resulting in substitution of a glycine by aspartic acid. **A**, DNA sequencing chromatograms of exon 69 in *Col7a1* of wild-type (left panel) and homozygous mutant (right panel) rats. The arrows indicate the G to A mutation (c.G5600A). **B**, The mutation leads to a codon change resulting in a glycine to aspartic acid substitution (p.G1867D). **C**, The mutated exon 69 codes for a stretch of the collagenous domain, N-terminally from the non-collagenous hinge region of C7. **D**, Both the mutated base and the amino acid are conserved in humans (ENST00000328333), suggesting that a similar mutation could result in human DEB.

The above evidence suggested that C7 was abnormal in the affected rats. Therefore, we performed mutation analysis of the *Col7a1* genomic DNA. Sequencing revealed a G to A substitution in base 5600 in exon 69 (ENSRNOT00000027994; GenBank accession number KC834559) (Figure 3A), which leads to substitution of a glycine residue with aspartic acid at position 1867; p.G1867D (Figure 3B). Exon 69 codes for a stretch of the collagenous domain close to the so-called “hinge region” of C7 [23] (Figure 3C). The collagenous domain is highly conserved between species (Figure 3D), suggesting that this glycine substitution could also cause human DEB. However, the corresponding mutation has not yet been reported in DEB patients (HGMD database: http://www.biobase-international.com/product/hgmd) and [9].

### Mode of inheritance

Human studies have shown that glycine to aspartic acid substitutions within the C7 collagenous domain can give rise to DEB of both dominant and recessive mode of inheritance [9], [10]. In the present rat colony, a spontaneous founder mutation has occurred. In order to firmly establish the mode of inheritance, wild-type rats were crossed with homozygous carriers of the mutation. For fast genotyping a restriction enzyme digestion strategy utilizing the fact that the c.G5600A mutation abolishes a Hpy8I restriction site was used (Figure S1).

**Figure 4:**
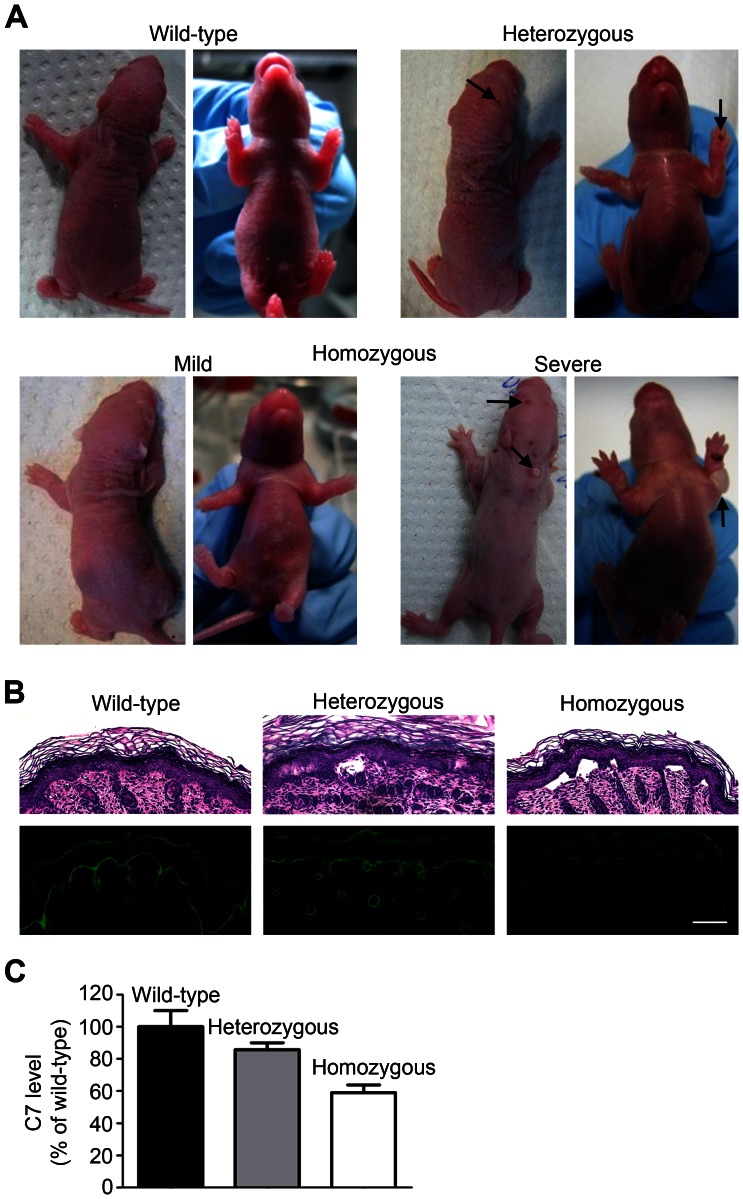
Dominant inheritance and gene-dosage effect of the mutation. **A**, Two-day-old rat pups, wild-type, heterozygous or homozygous carriers of the p.G1867D mutation. The heterozygous pups were generated from breeding wild-type males with homozygous females. Heterozygous rats display blistering of paws and back skin (arrows); the phenotype is milder than that of the homozygous pups (gene-dosage effect). **B**, H&E and immunofluorescence staining of the skin of newborn wild-type, heterozygous or homozygous rats. Note the limited dermal-epidermal separation in heterozygous skin, but extensive separation in homozygous skin. The mutation leads to a reduction in the C7 content at the DEJZ, as seen by C7 immunostaining. The reduction is more pronounced in homozygous animals. Scale bar = 100 µm. **C**, Semiquantification of C7 staining in **B**.

Breeding wild-type with homozygous p.G1867D rats resulted in heterozygous pups that displayed a skin blistering phenotype. However, the phenotype was much milder than in the homozygous carriers (Figure 4A). This determined a dominant mode of inheritance, and therefore, the rats represent a model of DDEB. Additionally, there is a gene-dosage effect with homozygous rats being more strongly affected, a phenomenon which can also be observed in families with other subtypes of EB [24].

Morphological analysis of the skin of the homozygous pups showed spontaneous, extensive blistering (Figure 4B). Semiquantitative immunofluorescence staining demonstrated reduction of C7 content at the DEJZ; this was stronger than in heterozygous animals (Figure 4B and C), thus supporting a gene-dosage effect.

Interestingly, the homozygous pups exhibited a variable phenotype (Figure 4A), suggesting involvement of disease modifier genes. Environmental factors seem to play a lesser role in this specific setting, since rats were inbred and kept under similar conditions.

### C7 expression and stability

**Figure 5:**
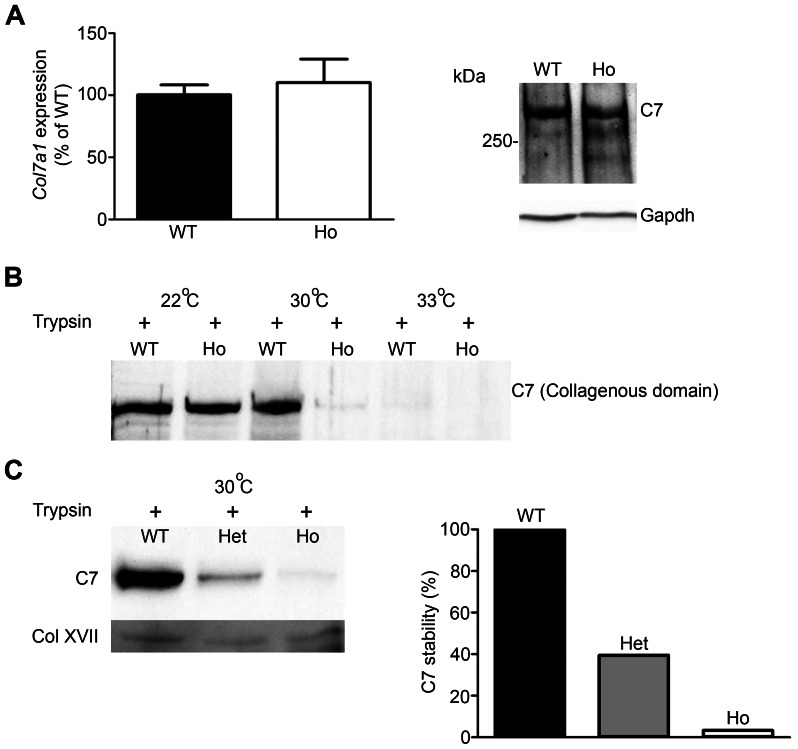
The p.G1867D mutation impacts protein stability. **A**, C7 expression in cultured fibroblasts from wild-type and p.G1867D homozygous rats. qPCR analysis (left panel) and Western blot (right panel) demonstrate no notable change in mRNA or protein expression. **B**, Limited trypsin digestion reveals greatly reduced thermal stability of mutant C7, as shown by Western blot detecting the intact collagenous domain of C7. Mutant C7 containing the p.G1867D substitution was degraded at lower temperature than wild-type C7, indicating a reduction in thermal stability due to faulty triple helix folding of the collagenous domain. WT = wild-type; Ho = homozygous for p.G1867D. **C**, Limited trypsin digestion of C7 extracted from wild-type, heterozygous and homozygous keratinocytes. The lysates were heated to 30°C and allowed to cool down to room temperature before limited trypsin digestion. Western blot detecting the intact collagenous domain of C7 (left panel) shows reduced stability of heterozygous molecules and loss of stability of homozygous molecules. The right panel shows densitometric quantification of the collagenous domain of C7 and normalization to collagen XVII, which was used as a loading control. The value of wild-type C7 was set to 100% and the percent stability, as indicated by resistance to trypsin digestion, of heterozygous and homozygous C7 was calculated in relation to this value. WT = wild-type; Het = heterozygous for p.G1867D; Ho = homozygous for p.G1867D.

The electron microscopy and the immunofluorescence data pointed to poor structure of anchoring fibrils and a slight reduction of C7 in the skin of affected animals. In order to determine the causes, we analyzed C7 expression and biochemical stability in cultured keratinocytes and fibroblasts. C7 expression at the mRNA or protein level was not changed in mutant rat skin (Figure 5A and data not shown).

Glycine substitutions in the C7 collagenous domain are known to impact triple helix folding and to decrease the stability of the molecule [25], [26]. Therefore, we investigated the C7 stability by limited trypsin digestion. Correctly folded collagenous domains show resistance to limited trypsin digestion, whereas misfolding increases sensitivity. When exposed to a temperature gradient followed by short trypsin digestion, C7 with the p.G1867D substitution became degraded at lower temperature than wild-type C7 (Figure 5B and C). This indicates improper folding of the collagenous domain, reduced stability and increased susceptibility to tissue proteinases, which is the underlying cause of reduced C7 and anchoring fibril content at the DEJZ in mutant rats. These findings also support a gene-dosage effect of the mutation, as homozygous C7 molecules containing only mutated C7 α-chains were less stable than C7 molecules composed of wild-type and mutant α-chains (Figure 5C).

## Discussion

Here we characterize a novel rat model of DDEB. In comparison to mice, rats' larger body and litter size, and feeble temper, make them more attractive to work with. Consequently, rats are the model of choice in many aspects of drug development, including studies of drug safety and metabolism [27]–[29]. The DDEB rat model disclosed here will be valuable for future therapy development, but also for studies on disease mechanisms in DEB and on C7 biology.

The mutation inherited in a dominant manner, yet there is also a clear gene-dosage effect of the mutated C7, a phenomenon which has also been described in families affected by other EB subtypes [24]. At lower dosages (in heterozygotes) the mutated C7 resulted in a milder phenotype, whereas at higher dosages (in homozygotes) the phenotype was more severe. Interestingly, also the homozygous DDEB rats displayed variable phenotypes. This replicates well the situation of DDEB patients, where the same mutation can give rise to a wide spectrum of clinical presentations [8]. These observations emphasize the probable role of modifier genes in DDEB pathogenesis, as the inbred rats were held under similar environmental conditions. Given that very little is known about modifier genes in DEB [10], [30], this rat model offers a valuable tool for future studies on genes that regulate disease severity. Identification of such genes will be important for design of biologically valid therapeutic strategies.

The rat model presented here is the first viable small animal model of DDEB, and also the first rat model of DEB. The phenotype is caused by a glycine substitution mutation, which is of importance, as 25% of all C7 mutations reported are glycine substitutions (HGMD database: http://www.biobase-international.com/product/hgmd). All previous DEB mouse models are transgenic, with either the entire *Col7a1* gene deleted, reduced expression of C7, or the combination of *Col7a1* deletion and forced expression of mutated human C7 [11]–[13]. Since the DDEB rat has a spontaneous mutation, it more closely resembles DEB patients. Furthermore the DDEB rat shows a gene-dosage effect and phenotype variability, which could assist in the delineation of disease modifying genes. This model will be a useful tool for studying novel therapeutic strategies in dominantly transmitted DEB, such as gene silencing, chemically-induced exon read-through [31] or splice modulation [32].

## Materials and Methods

### Animals

The rats on a Sprague Dawley background were originally housed at the Max Delbrück Center for Molecular Medicine, Berlin, where the phenotype, which had developed spontaneously, was first observed. Later breeding pairs of affected rats were transferred to the Center for Experimental Models and Transgenic Services at the University of Freiburg. All animal work was performed in strict compliance with the German federal animal welfare law (“Tierschutzgesetz”, Paragraph 4) and after approval through the official animal welfare officer (“Tierschutzbeauftragter”) of the CEMT, University Freiburg (IACUC). The rats were given food and water ad libitum. After birth, affected litters were monitored and photographed every day for two weeks and then once every week. Wild-type Sprague Dawley rats used to generate heterozygotes were purchased from Charles River (Sulzfeld, Germany).

### Light microscopy

Skin specimens were fixed in 10% formalin for H&E staining or snap-frozen in optimal cutting temperature compound on dry-ice for analysis of C7 expression. The fixed skin was embedded in paraffin, sectioned, deparaffinized, rehydrated and H&E stained using standard procedures. Snap frozen material was cut on a cryotome. Before staining, the optimal cutting temperature compound was washed off from the slides in PBS and slides fixed in ice-cold acetone. The slides were blocked in 3% BSA-PBS-T, after which they were stained with a rabbit polyclonal antibody to human placental C7 (Calbiochem Merck, Darmstadt, Germany). After washing and incubation with Alexa 488 conjugated secondary antibodies, the slides were counterstained with DAPI to visualize nuclei, washed and mounted. Images of H&E and C7 stainings were acquired with a Zeiss Axiocam mounted in a Zeis Axiovision microscope (Carl Zeiss, Jena, Germany). Images were further processed using the Zen2010 software (Carl Zeiss) and quantified with the Image J software (NIH, Bethesda, MD).

### Electron microscopy

For transmission electron microscopy, skin of 4-day-old rats was fixed in 4% paraformaldehyde and 2% glutaraldehyde. After fixation it was washed twice in 0.1 M cacodylate buffer, and incubated for 1 h in 1% osmium tetroxide solution. Following dehydration in ethanol and propylene oxide, samples were embedded in an epoxide resin. Sections of 70 nm thickness were mounted on microscopy grids and stained with 5% uranyl acetate and Reynold's solution. Quantification of randomly selected areas was performed using the Image J software (NIH).

### Cell culture

Keratinocytes and fibroblasts were isolated and cultured as previously described [12]. Shortly, 1- to 3-day-old rat pups were killed by decapitation. The skin cleaned with ethanol, removed and placed in 10% antibiotic-antimycotic in PBS (Gibco Invitrogen, Darmstadt, Germany). Under a sterile hood the skin was placed in a 6-well plate containing 0.25 mg/ml dispase diluted in DMEM:F12 (Stemcell, Grenoble, France) and incubated for 3 h at 37°C until the epidermis was readily separable from the dermis. The epidermis was then stripped from dermis with forceps and used for cell isolation. For keratinocyte isolation the epidermis was cut into small pieces and incubated with 0.5% trypsin for 10 min, trypsin was inactivated with serum, cells pelleted, washed in PBS and plated in CnT57 medium with supplements (CELLnTEC, Bern, Switzerland). Keratinocytes were passaged using TrypLE™ (Gibco Invitrogen). For fibroblast isolation, the dermal part was incubated with 500 units/ml collagenase type 1 (Worthington Biochemical Corporation, Lakewood, NJ) at 37°C for 1 h, passed through a 60 µm cell strainer. The cells were pelleted, washed in DMEM:F12 containing 10% FCS (Gibco Invitrogen) and plated in the same medium.

### DNA sequencing and PCR analysis

Genomic DNA was isolated by shortly boiling fibroblasts in 50 mM NaOH, neutralized with 0.1 volume 1 M Tris-HCl pH 7.4, cleaned by isopropanol precipitation and dissolved in double distilled water. Sequencing primers used for the *Col7a1* coding region of exons 68–70 and flanking introns were: forward 5′ GAAAAGGGAGATTCGGGTGT and reverse 5′ AATGGCACTTCAGGAAGCAT. PCR products were directly sequenced in an ABI 3100 genetic analyzer (ABI, Darmstadt, Germany). The mutation was deposited in GenBank, accession number KC834559.

The above primers were also used for genotyping using Hyp8I digestion. PCR products were directly digested with Hyp8I (Fermentas Thermo Fisher Scientific Inc., Waltham, MA) after amplification and analyzed on 1.5% agarose TBE gels.

For quantitative real-time PCR, mRNA from keratinocytes and fibroblasts was isolated using a RNA isolation kit (Macherey-Nagel, Dueren, Germany) following the manufacturer's instructions. The RNA was transcribed to cDNA (Fermentas Thermo Fisher Scientific Inc). Quantitative real-time PCR using SYBR Green labeling was run on a CFX-96™ Real-Time system (Biorad, Carlsbad, CA). Primers used were: *Col7a1* forward 5′GTGGCCATTGAAGAGCTAGG and reverse 5′TTCCCTTCAGGTCCAGACAC; *Gapdh* forward 5′ TTGATGGCAACAATCTCCAC and reverse 5′ CGTCCCGTAGACAAAATGGT.

### Western blotting and limited trypsin digestion assay

Cells were extracted with NP-40 lysis buffer [12]. Lysates were boiled in sample buffer containing 8 M urea, separated on 7% SDS-Polyacrylamide gels and electrotransferred onto nitrocellulose membranes. The membranes were blocked in 5% milk in TBS-T, incubated with primary antibodies (either rabbit polyclonal antibody to human placental C7, Calbiochem) or mouse monoclonal glyceralaldehyde-3-phosphate dehydrogenase (GAPDH) antibody (Billerica, MA, USA) in blocking buffer, washed and probed with HRP-conjugated secondary antibodies. The blots were developed with ECL (Fermentas Thermo Fisher Scientific Inc.) and detected using a chemiluminiscence detection system (Peqlab, Erlangen, Germany).

For the limited trypsin digestion assay to assess C7 stability [33], [34], rat keratinocytes were grown to confluence. Then 50 µg/ml ascorbic acid was freshly added every day, for 5 days. The cell layer and extracellular matrix were extracted in NP-40 buffer. Lysate aliquots were heated for 1 min, allowed to cool down to room temperature for 10 seconds and trypsin (Serva, Heidelberg, Germany) to a total volume of 0.1% v/v trypsin was added to the protein lysates and the lysates were incubated for 30 seconds before the reaction was stopped. The samples were then analyzed by Western blotting. The C7 collagenous domain was detected using the rabbit polyclonal antibody H-120 (Santa Cruz Biotechnology Inc., Santa Cruz, CA) and collagen XVII with rabbit anti-human collagen XVII [35].

## Supporting Information

Figure S1 **Restriction enzyme-mediated genotyping.** Exons 68–70 of the *Col7a1* gene were amplified by PCR, and the PCR product digested with the restriction enzyme Hyp8I. The c.G5600A substitution abolishes a Hyp8I cleavage site in exon 69. Thus, digestion of the wild-type *Col7a1* DNA results in two cleavage products of similar size, whereas DNA carrying the c.G5600A substitution is not digested by Hyp8I. + = digestion with Hyp8I, − = control without digestion enzyme.
(TIF)
