# Supplementary figures and images for: Rat Model for Dominant Dystrophic Epidermolysis Bullosa: Glycine Substitution Reduces Collagen VII Stability and Shows Gene-Dosage Effect
Source: PLoS One. 2013 May 23;8(5):e64243. doi: 10.1371/journal.pone.0064243 (PMC3662756; doi:10.1371/journal.pone.0064243)

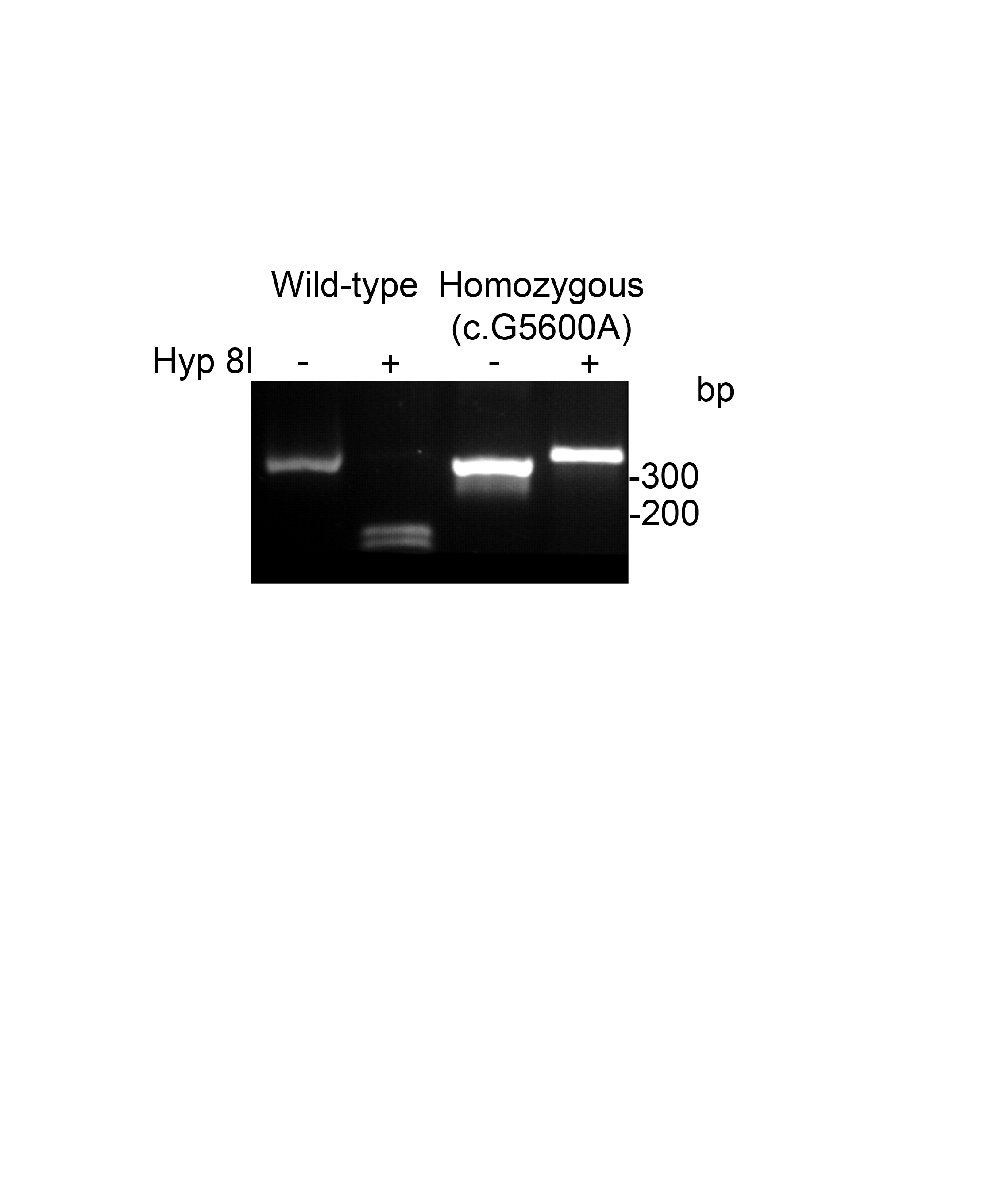

Supplement: Figure S1 — Restriction enzyme-mediated genotyping. Exons 68–70 of the Col7a1 gene were amplified by PCR, and the PCR product digested with the restriction enzyme Hyp8I. The c.G5600A substitution abolishes a Hyp8I cleavage site in exon 69. Thus, digestion of the wild-type Col7a1 DNA results in two cleavage products of similar size, whereas DNA carrying the c.G5600A substitution is not digested by Hyp8I. + = digestion with Hyp8I, − = control without digestion enzyme. (TIF) [file pone.0064243.s001.tif]
